# Les caractéristiques de la population couverte par le régime de l’assurance maladie obligatoire au Maroc

**DOI:** 10.11604/pamj.2018.30.266.13209

**Published:** 2018-08-08

**Authors:** Amal Yassine, Abdelkader Jalil El Hangouche, Naoufel El Malhouf, Siham Maarouf, Jamal Taoufik

**Affiliations:** 1 Laboratoire de Chimie Thérapeutique, Faculté de Médecine et de Pharmacie Rabat, Université Mohamed V, Maroc; 2 Agence Nationale de l’Assurance Maladie (ANAM), Rabat, Maroc; 3 Laboratoire de Physiologie, Faculté de Médecine et de Pharmacie de Rabat, Université Mohamed V, Maroc

**Keywords:** Assurance maladie obligatoire, affection de longue durée, ressources financières, Maroc, Compulsory health insurance, long duration disease, financial resources, Morocco

## Abstract

**Introduction:**

Au Maroc, le régime de l’Assurance Médicale Obligatoire (AMO) est entré en vigueur depuis l’année 2005. Cette couverture a débuté en premier, par les salariés des secteurs public et privé puis les étudiants pour s’étendre progressivement aux indépendants. L’objectif de cette étude est d’établir le profil de la population couverte par l’AMO au Maroc.

**Méthodes:**

Il s’agit d’une étude descriptive de la population couverte par l’AMO s’intéressant aux données recueillies auprès de l’Agence Nationale de l’Assurance Maladie Marocaine et les caisses de l’Assurance Maladie.

**Résultats:**

La population couverte par l’AMO était à fin 2014 de 8.428.218 bénéficiaires, soit 34% de la population générale. La population atteinte d’Affection de Longue Durée (ALD) ne dépasse pas 2,78% de la population couverte. La part des assurés actifs représente 81% de la population couverte. Dans le secteur privé, le salaire brut des affiliés est en moyenne entre 140 et 500$ par mois chez les actifs et moins de 280$ par mois chez les pensionnés. Alors que dans le secteur public, le salaire brut des affiliés est en moyenne entre 280 et 825$ par mois chez les actifs et entre 140 et 500$ par mois chez les pensionnés.

**Conclusion:**

La connaissance des caractéristiques de la population couverte par le régime de l’Assurance Maladie Obligatoire au Maroc est nécessaire pour assurer la régulation et la pérennité du système.

## Abstract (en)

**Introduction:**

in Morocco, Compulsory Medical Insurance (CMI) entered into force in 2005. Insurance first covered health expenses of employees in public and private sectors, then of students. It was gradually expanded to independent workers. This study aims to determine the profile of the population covered by CMI in Morocco.

**Methods:**

We conducted a descriptive study of the population covered by CMI based on data collected from the National Health Insurance Agency in Morocco and from the Health Insurance funds.

**Results:**

A total of 8.428.218 persons were covered by CMI at the end of 2014, reflecting a rate of 34% of the general population. People having long duration disease (LDD) did not exceed 2.78% of the population covered by CMI. Active insured accounted for 81% of the population covered. In the private sector, gross salary of active affiliates ranged, on average, between $ 140 and $ 500 per month while gross salary pensions was less $280 per month. In the public sector, gross salary of active affiliates ranged, on average, between $ 280 and $ 825 per month while gross salary pensions ranged between $ 140 and $ 500 per month.

**Conclusion:**

Knowledge of the characteristics of the population covered by Compulsory Health Insurance in Morocco is necessary to ensure regulation and sustainability in the insurance sector.

## Introduction

La couverture universelle consiste à ce que tout un chacun puisse accéder aux services de santé dont il a besoin, sans subir de difficultés financières lors de leur paiement. En progressant vers cet objectif, les gouvernements font face à trois questions fondamentales [1]: comment un tel système de santé est-il financé? Comment peuvent-ils protéger les gens des conséquences financières liées à la maladie et aux paiements des services de santé? Comment peuvent-ils encourager une utilisation optimale des ressources? Au Maroc, l’accès aux soins pour la population reste lié principalement au niveau de la couverture médicale et de l’offre de soins sur le territoire national; 24% de la population est confrontée à des difficultés à accéder aux soins de santé ce qui pose un problème de gouvernance du système de santé. Parmi les 22 pays et territoires de la région de l´Est-Méditerranée (OMS EMRO), le Maroc se classe parmi les cinq derniers concernant la composante de la santé des adultes et la composante des dépenses de la santé de l´indice de développement humain. En outre, les dépenses de santé directes ont atteint 88,3% (pourcentage des dépenses privées de santé) entre 2009 et 2013 [2]. A cet effet, la couverture médicale au Maroc s’est concrétisée par l’avènement de la loi n° 65-00 portant code de la couverture médicale de base (CMB) [3] et la nouvelle constitution de 2011 [4], qui permettent le développement de la solidarité nationale et l’accès équitable aux soins de santé à l’ensemble de la population. La loi 65-00 portant code de la couverture médicale de base (CMB) institue l’Assurance Maladie Obligatoire (AMO) de base et le Régime d’Assistance Médicale pour les économiquement démunis (RAMED) [3]. Le régime d’Assurance Médicale Obligatoire (AMO) est entré en vigueur depuis l’année 2005. Cette couverture a débuté en premier, par les salariés des secteurs public et privé pour s’étendre progressivement aux étudiants. Ce processus a connu une dynamique importante en matière d’affiliation et d’immatriculation des effectifs au cours de ces dernières années. Au Maroc, une partie des salariés des secteurs public et privé ne sont pas encore couverts par l’AMO en raison des dispositions transitoires de l’article 114 de la loi n°65-00. En effet, le législateur a accordé à cette population éligible à l’AMO et non encore couverte dénommée « population de l’article 114 » un délai de 5 ans renouvelable à partir de la publication des décrets réglementaires de la loi n°65-00 [3]. L’objectif de cette étude est d’établir le profil de la population couverte par l’AMO au Maroc.

## Méthodes

### Type de l’étude

Il s’agit d’une étude descriptive de la population couverte par le régime de l’’Assurance Maladie Obligatoire (AMO) visant à identifier et analyser les principaux paramètres qui déterminent le mode de couverture médicale de base de la population couverte par à l’Assurance Maladie Obligatoire (AMO) et celui de la population bénéficiant des dispositions de l’article 114, éligible à l’AMO et non encore couverte par le régime.

### Source de l’étude

Le recueil des données a été fait auprès de l’Agence Nationale de l’Assurance Maladie Marocaine, relatif aux populations couvertes par les différents régimes de l’Assurance Maladie Obligatoire (AMO). Ce recueil est fait après chaque année d’exercice comptable des organismes gestionnaires de l’AMO et allant de 2008 à 2014. Le recueil de données a respecté les dispositions réglementaires de la loi n°09-08 [5], relative à la protection des personnes physiques à l’égard du traitement de données à caractère personnel. Les données recueillies ne comprennent que les variantes économiques et épidémiologiques à étudier. L’exploitation des paramètres identifiés résulte de l’exercice des organismes gestionnaires de l’AMO entre la période entre 2008 et 2014. Seules les données validées par le conseil d’administration de l’Agence Nationale de l’Assurance Maladie ont été exploitées.

### Lieu de l’étude

L’Agence Nationale de l’Assurance Maladie (ANAM) du Maroc et la faculté de médecine et de pharmacie de Rabat, Maroc.

### Critères d’inclusion et d’exclusion

Dans notre étude, l’effectif retenu a été celui des bénéficiaires ayant les droits ouverts à l’Assurance maladie. Les bénéficiaires ayant eu la fermeture de droit à l’Assurance Maladie ou un statut indéterminé ont été exclus de l’étude.

### Les paramètres étudiés

Effectif de la population couverte par l’AMO; Le Profil des bénéficiaires de l’AMO: actif versus pensionné avec les ayants droits, age du bénéficiaire, population porteuse de l’Affection de Longue Durée (ALD). Le secteur d’activité; la répartition géographique; les ressources financières. Notons que: 1) L’actif est défini comme une personne en activité professionnel avec ses ayants droits (conjoint (s) et les enfants); 2) Le pensionné est défini comme une personne à la retraite avec ses ayants droits. Les pensionnés regroupent les personnes à la retraite ou en invalidité, les conjoint (s) survivant (s) et les ayants droits (enfants). 3) La population porteuse d’ALD est l’ensemble des bénéficiaires enregistrés auprès des organismes gestionnaires de l’AMO pour au moins une pathologie chronique.

### Limites de l’étude

Le processus de l’affiliation et l’immatriculation aux caisses de l’assurance maladie obligatoire est très dynamique. En effet pour chaque année le flux d’entrée et de sortie de la population couverte par l’assurance maladie au régime est variable, il est dû au changement permanent du statut et d’activité professionnel et social des bénéficiaires (changement de secteur d’activité, mariage, divorce, invalidité et décès …), ce qui rend le recueil et l’exploitation des données hétérogènes d’une année à une autre.

## Résultats

**Figure 1:**
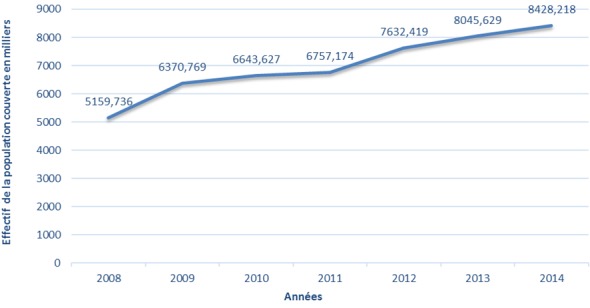
Évolution de la population couverte par le régime AMO entre 2008 et 2014 en milliers

**Figure 2:**
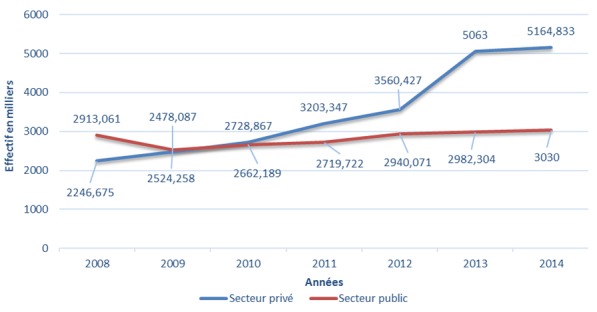
Évolution de la population couverte par le régime AMO en fonction du secteur entre 2008 et 2014 en milliers

**Figure 3:**
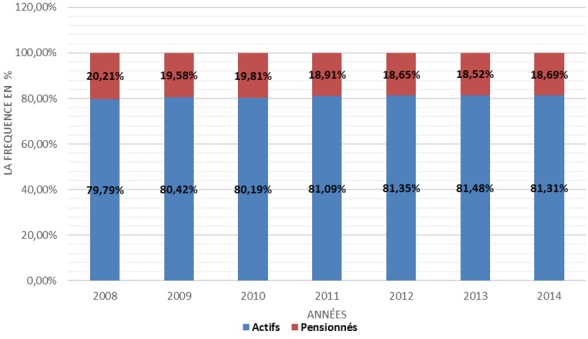
Répartition de l’effectif AMO en fonction du type d’assuré entre 2008 et 2014

**Figure 4:**
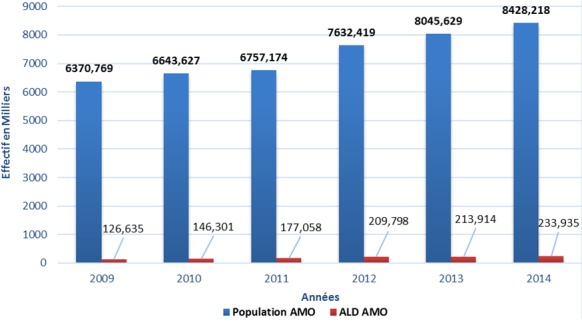
Évolution de l’effectif de la population en fonction de l’ALD entre 2009 et 2014 en milliers

La population couverte par le régime AMO de base a évolué au cours des sept ans passant de 5.159.736 en 2008 à 8.428.218 bénéficiaires en 2014, soit une évolution moyenne de 7,66% (Figure 1). Pour le secteur privé, celle-ci est passée de 2.246.675 en 2008 à 5.164.833 bénéficiaires en 2014, soit une évolution moyenne de 12,5% sur la même période alors que la population couverte du secteur public est passée de 2.913.061 en 2008 à 3.030.000 bénéficiaires en 2014, soit une évolution moyenne de 0,40% (Figure 2). La part de la population couverte des actifs reste supérieure à celle des pensionnés entre 2008 et 2014 (Figure 3). Pour le secteur privé, la tranche d’âge chez les actifs (assurés et ayant droit) est prédominante chez la population de moins de 45 ans alors chez les pensionnés, celle-ci est au-delà de 70 ans. Pour le secteur public, la tranche d’âge chez les actifs (Assurés et ayants droit) est entre 30 et 60 ans alors que chez les pensionnés, celle-ci entre 60 ans et 70 ans. La population atteinte de maladie chronique ou porteuse d’Affection de Longue Durée (ALD) représente une part très faible par rapport au reste de la population couverte avec une fréquence de 2,8% en 2014 (Figure 4).

**Figure 5:**
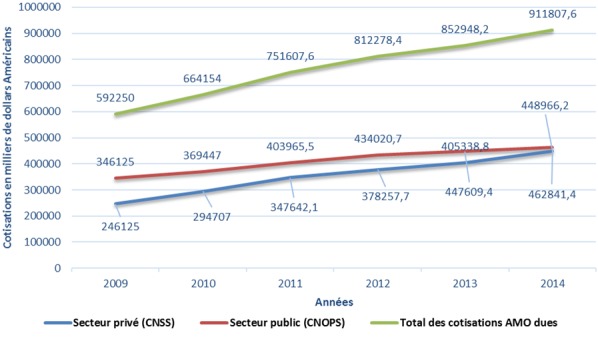
Évolution des cotisations dues des deux secteurs public et privé entre 2009 et 2014 en milliers de Dollars américains

**Figure 6:**
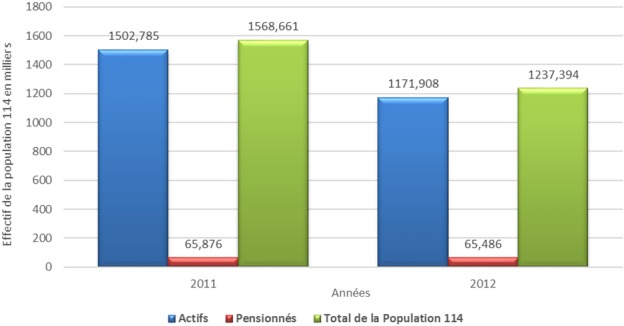
Évolution de la population 114 du secteur privé entre 2011 et 2012 par type d’assuré en milliers

**Figure 7:**
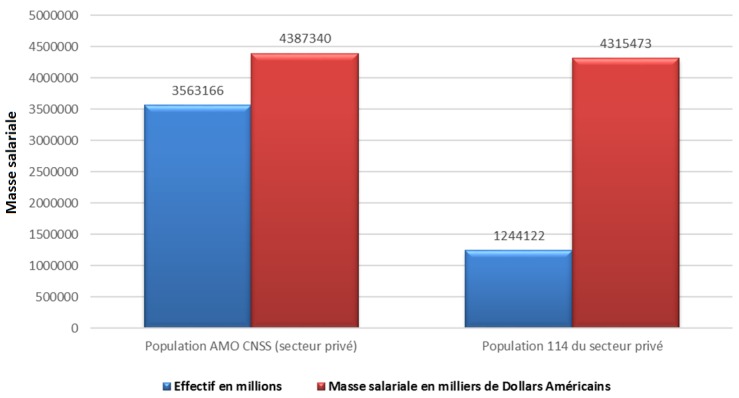
Comparaison entre la population AMO du secteur privé et la population 114 privée en fonction de l’effectif et la masse des salaires pour l’année 2012

La population couverte du secteur privé se concentre fortement dans la région du Grand Casablanca (25,96%), suivie de la région Souss Massa Draa (12, 95%) alors que pour le secteur public, les assurés se concentrent principalement à la région de Rabat Salé, Zemmour Zear (19,61%) suivi de la région du grand Casablanca (13,29%). Dans le secteur privé, le salaire brut des affiliés est en moyenne entre 140 et 500 $ par mois chez les actifs et de moins de 280$ par mois chez les pensionnés. Alors que dans le secteur public, le salaire brut des affiliés est en moyenne entre 280 et 825$ par mois chez les actifs et entre 140 et 500 $ par mois chez les pensionnés. L’évolution des cotisations des assurés de l’AMO est en croissance avec un écart entre les deux secteurs. Le secteur public a plus de contributions par rapport au secteur privé (Figure 5). La population éligible et non encore couverte par l’AMO du secteur privé (population de l’article 114) représente 1 237 394 personnes. Son effectif est en diminution constante depuis 2011 à titre d’exemple, cette population a baissé de 26,7% de 2011 à 2012 (Figure 6). En comparant la masse des salaires déjà affiliés à l’AMO du secteur privé et la population éligible et non encore couverte (population de l’article 144), la différence de masse de salaires est seulement de 1,64% pour un écart d’effectif de 2 323 033 bénéficiaires (Figure 7). La population bénéficiant des dispositions de l’article 114 du secteur public (population éligible et non encore couverte par l’AMO) est estimé à 140 154 bénéficiaires en 2014.

## Discussion

Au Maroc, la Couverture médicale de base est un programme politiquement et socialement stratégique pour le pays, il représente un élément fondamental du développement de la solidarité nationale et de l’accès équitable de toute la population Marocaine aux soins de santé. L’engagement de l’Etat est en effet, d’assurer l’accès aux soins à toutes les couches sociales de la population par la prise en charge solidaire des dépenses de santé, de garantir une offre de soins de qualité répartie d’une manière harmonieuse sur le territoire Marocain, et de relever les défis afin d’atteindre la couverture sanitaire universelle. La population couverte par le régime de l’Assurance Maladie Obligatoire (AMO) présente plusieurs caractéristiques et propriétés avec un profil de consommation médicale pouvant influencer l’équilibre financier des caisses d’assurance maladie et la pérennité des régimes de couverture médicale. L’analyse des différentes variantes de la population couverte apporte des résultats qui permettent la possibilité de comprendre la dynamique de la couverture médicale au Maroc.

Dans notre étude l’évolution de la population couverte par le régime de l’AMO s’explique par la dynamique d’affiliation et d’immatriculation auprès des organismes gestionnaires de l’AMO des salariés des deux secteurs. Cette dynamique est due entre autre à la croissance économique du pays, la croissance du PIB du Maroc était de 4,5% en 2015 (PIB Maroc est à 1.006 milliard de dollars) avec un taux d’inflation de 1,6% en 2016 [6] et à l’embauche continue des salariés. La variation de la dynamique de la croissance de la population couverte entre les secteurs public et privé s’explique par la taille du secteur privé, le nombre croissant des entreprises privées implantées sur le sol Marocain et la diversification des secteurs d’activité, un rythme d’embauche accéléré (affiliation et immatriculation) et le basculement continue de la population qui bénéficient des dispositions de l’article 114 du secteur privé au régime d’Assurance Maladie de Base.

La pyramide d’âge de la population couverte connait une différence entre les secteurs privé et public et par type d’assuré. Pour le secteur privé, celle-ci se caractérise par une base très large due à la prédominance de la population jeune des actifs. La tranche d’âge de la population des pensionnés est concentrée essentiellement au-delà de 70 ans, cela peut s’expliquer par la tendance de la population du secteur privé à garder son statut actif même après l’âge légal de la retraite et ne devenir pensionné à partir de 70 ans. Pour le secteur public, les actifs qui sont moins jeunes et cela s’explique probablement par la stabilité de la population du secteur. La tranche d’âge des pensionnés se caractérise par une prédominance entre 60 et 70 ans et cela pourrait s’expliquer par le changement de statut de cette population, *actif devient pensionné*, à l’âge légal de la retraite (60 – 65 ans) [7]. La concentration de la population couverte du secteur privé autour de la capitale économique s’explique par l’implantation massive des entreprises privées à forte activité économique et l’activité de la pêche maritime dans cette région [8]. Pour le secteur public, la concentration de la population couverte est autour de la capitale administrative du Maroc où il y a un nombre important d’administrations publiques.

La différence des salaires entre les secteurs public et privé s’explique par le fait que: 1) Les salaires dans le secteur public sont réglementés et fixés pour la fonction publique [9]. L’avancement en grade et échelons des fonctionnaires est fixé par voie réglementaire. Toutefois dans le secteur privé, les salaires sont soumis aux négociations entre l’employeur et l’employé. Ils sont variables entre les salariés du même profil académique ou professionnel, ils dépendent également de la taille de l’entreprise et sa capacité de générer des profits. Les avancements sont également en fonction de l’ancienneté de l’employé et restent soumis aux négociations. 2) La dynamique de fermeture de droit [4] à l’AMO chez la population du secteur privé est beaucoup plus importante que pour le secteur public. Ceci est dû aux arrêts fréquents d’activité dans le secteur privé et à la sous déclaration des employeurs de certaines entreprises de l’ensemble de leurs employés. Par conséquent, cette catégorie ne cotise pas. 3) La population bénéficiant des dispositions de l’article 114 représente une grande masse salariale et n’a encore pas totalement basculé à l’AMO du secteur privé.

La différence des cotisations dues, entre les deux secteurs, est marquée par la contribution élevée du secteur public durant la période 2009 - 2013. Cette variation s’explique par: 1) La tranche des salaires est plus importante dans le secteur public avec un effectif plus bas. 2) Le taux de cotisation des actifs est de 5% du secteur public versus 4% du secteur privé [10]. Il est à noter que le taux de cotisation en secteur privé a augmenté à partir de 2016 pour un taux de 4,56% [11]. Le secteur public a une cotisation plafonnée entre 7 et 40 Dollars Américains et un taux de cotisation de 2,5% pour les [12].

Pour la population bénéficiant des dispositions de l’article 114 de la loi 65-00, la proportion importante des actifs par rapport aux pensionnés s’explique probablement par le fait que les entreprises privées inscrivent leurs salariés auprès des assurances privées via un contrat de groupe [13], tant que le salarié est en statut actif, une fois pensionné, il bascule à l’AMO du secteur privé. Si les écarts d’effectif entre la population couverte par l’AMO du secteur privé et celle bénéficiant des dispositions de l’article 114 sont importants, les masses salariales des deux catégories sont en revanche, pratiquement similaires. Ceci en raison des salaires élevés chez la population relevant de l’article 114. La population hors couverture de l’AMO du secteur public est faible par rapport à la population déjà couverte par le même régime.

## Conclusion

La performance du système de couverture médicale obligatoire de base au Maroc passe par l’identification des variantes qui le composent, la compréhension du profil de la population couverte et le mode de financement du régime. Sa pérennité nécessite l’augmentation des ressources financières des organismes gestionnaires du régime de l’Assurance Maladie de Base et la veille à l’équilibre financier pour favoriser l’accès aux soins des populations et mieux financer le système de santé. L’augmentation des ressources financières de l’AMO nécessite d’intégrer la population éligible et non encore couverte ainsi que l’extension de la couverture médicale aux autres catégories socio-professionnelles « les libéraux » ainsi que le relèvement du plafond des cotisations (déplafonnement des cotisations) pour pouvoir augmenter les recettes du secteur public.

### Etat des connaissances actuelles sur le sujet

- L’entrée en vigueur du régime de l’Assurance Maladie Obligatoire au Maroc;
- Le financement de la santé au Maroc.

### Contribution de notre étude à la connaissance

- Identifier et analyser des caractéristiques de la population couverte par l’Assurance Maladie Obligatoire au Maroc;
- Connaitre le mode de financement et les ressources financières du régime de l’AMO entre les secteurs public et privé.

## Conflits d’intérêts

Les auteurs ne déclarent aucun conflit d’intérêts.
