# Corrigendum: Les caractéristiques de la population couverte par le régime de l’assurance maladie obligatoire au Maroc

**DOI:** 10.11604/pamj.2019.33.173.19359

**Published:** 2019-07-04

**Authors:** Amal Yassine, Abdelkader Jalil El Hangouche, Naoufel El Malhouf, Siham Maarouf, Jamal Taoufik

**Affiliations:** 1 Laboratoire de Chimie Thérapeutique, Faculté de Médecine et de Pharmacie Rabat, Université Mohamed V, Maroc; 2 Agence Nationale de l’Assurance Maladie (ANAM), Rabat, Maroc; 3 Laboratoire de Physiologie, Faculté de Médecine et de Pharmacie de Rabat, Université Mohamed V, Maroc

**Keywords:** Compulsory health insurance, long duration disease, financial resources, Morocco, corrigendum, Compulsory health insurance, long duration disease, financial resources, Morocco

## Abstract

Ce Corrigendum modifie l'article original «Les caractéristiques de la population couverte par le régime de l'assurance maladie obligatoire au Maroc». The Pan African Medical Journal. 2018;30:266. doi:10.11604/pamj.2018.30.266.13209.

## Abstract (en)

This corrigendum modifies the original article: The characteristics of the population covered by Compulsory Health Insurance (CMI) in Morocco. The Pan African Medical Journal. 2018;30:266. doi:10.11604/pamj.2018.30.266.13209.

## Corrigendum

Dans l'article original archivé sur Pubmed, le nom de l'auteur Abdelkader Jalil El Hangouche est archivé Hangouche AJE au lieu de El Hangouche AJ [1].

## References

[1] Yassine A, Hangouche AJE, Malhouf NE, Maarouf S, Taoufik J (2018). Les caractéristiques de la population couverte par le régime de l'assurance maladie obligatoire au Maroc. Pan Afr Med J.

